# Isolation, differentiation and biodiversity of ureolytic bacteria of Qatari soil and their potential in microbially induced calcite precipitation (MICP) for soil stabilization

**DOI:** 10.1039/c7ra12758h

**Published:** 2018-02-06

**Authors:** Shazia Bibi, Meriam Oualha, Mohammad Yousaf Ashfaq, Muhannad T. Suleiman, Nabil Zouari

**Affiliations:** Department of Biological and Environmental Sciences, College of Arts and Sciences, Qatar University PO Box 2713 Doha Qatar Nabil.Zouari@qu.edu.qa +974-4403-4559; Department of Civil and Environmental Engineering, Lehigh University USA

## Abstract

Biomineralization plays a key role in modifying the geological properties of soil, thereby stabilizing it against wind erosion, especially in areas characterized by harsh weather and harsh soil (calcareous and arid); *i.e.* Arabic Gulf region. Among soil microorganisms, ureolytic bacteria are capable of modifying soil characteristics and thus, inducing biomineralization. This research investigated the occurrence and diversity of ureolytic bacteria in Qatari soils, specifically to study their acquired potential to adapt to harsh conditions exhibiting ureolytic activity. Soil samples were collected from various locations in Qatar and were used to isolate the indigenous ureolytic bacteria. It was noticed that most of the ureolytic bacteria in Qatari soil belong to the genus *Bacillus* mainly *Bacillus cereus*. Identification and differentiation of 18 ureolytic isolates were performed using MALDI-TOF MS techniques while ribotyping (16S rRNA) molecular technique was used mainly for 6 selected strains. This study not only shows the diversity of species of ureolytic bacteria in Qatari soil but also shows the diversity in their protein profiles, which confirms that bacteria have adapted well to the harsh environment. In addition, the strains were evaluated based on a newly modified screening method in this work; *i.e.* production of arbitrary urease activity (AUA). Thus, the strains showing the highest AUA, exhibited the highest capability to produce urease enzymes induced by urea. Analysis of calcium carbonate precipitation utilizing SEM-EDX showed that the ureolytic bacteria also play a significant role in the precipitation of minerals such as CaCO_3_, in the presence of urea in soil. Therefore, this research showed a high occurrence of indigenous *Bacillus* bacteria in Qatari soil that can perform biomineralization and thus can be helpful, if properly stimulated, in enhancing soil stabilization, and for other local applications as well, since they are adapted to these soil and weather conditions.

## Abstract 2

Biomineralization plays a key role in modifying the geological properties of soil, thereby stabilizing it against wind erosion, especially in areas characterized by harsh weather and harsh soil (calcareous and arid); *i.e.* Arabic Gulf region.

## Graphical abstract

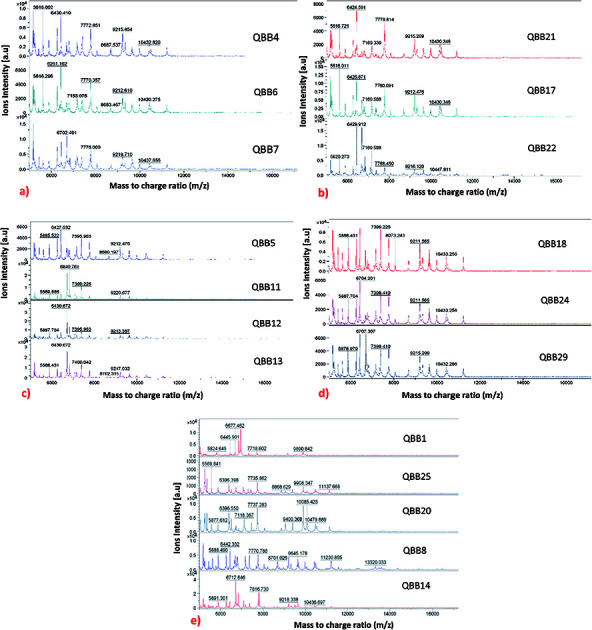

## 1. Introduction

The soil stabilization techniques currently available to minimize or avoid airborne particles due to wind erosion use significant amounts of fossil fuel energy and/or hazardous materials such as mixing techniques using bituminous materials as stabilizing agents. Investigating sustainable biological soil stabilization methods to resist soil erosion due to wind is more appealing, in particular, if the biological activities are delivered by indigenous soil bacteria living under the corresponding environmental conditions. Bio-modification methods are innovative soil stabilization improvement solutions that use indigenous bacteria in the soil to mimic and engineer naturally occurring processes.

Microorganisms play an important role in inducing the precipitation of minerals, hence, promoting a change in the geological properties of the soil or any earth component. Biomineralization can help to improve soil quality by increasing soil stiffness through the production of calcite precipitates using bacterial activities.^21^ Bacteria are important in biomineralization processes with, approximately, 200 different types known to induce calcium carbonate formation.^4^ There are two mechanisms known through which biomineralization occurs.^3^ Either, the bacterial cells provide nucleation site for the precipitation of calcium carbonates or ureolysis increases alkalinity of the surrounding environment resulting in precipitation. An alternative mechanism involves extracellular macromolecules; extracellular polymeric substances (EPS).^19^ EPS assists the microbial cell to adhere to different substrates while providing additional protection, nutrition and adsorption. EPS helps bacterial cells trap and precipitate sediments and minerals. After adhering to different surfaces, the bacterial cells start to dissolve the nutrients to obtain energy, resulting in precipitation of minerals as by-product. Ureolytic bacteria increases the pH through hydrolysis of urea and thus precipitates minerals.^15^*Bacillus* is the most common genus of bacteria known to perform biomineralization by implementing ureolytic activities. Some specific species are *B. sphaericus*,^7^*B. lentus*,^17^*B. pasteurii*^30^ and *B. licheniformis*.^16^

Bacteria involved in biomineralization are important in stabilizing soil, especially in regions and cities with windy and desert-like conditions such as in the Gulf region. Among large number of applications of ureolytic bacteria, the stabilization of soils, especially calcareous ones, is highly recommended. At such conditions, calcification may be favorable due to the stress by the elevated temperature and high calcium content in the soil. To assist biomineralization of calcite, bacterial cells must produce either constitutive or inducible urease, however, very few bacterial species produce constitutive urease.^5^ Only one bacterium, *Sporosarcina koreensis*, was isolated from the Qatari desert dunes and is shown to harbor the urease gene, as per its whole-genome sequencing.^1^ The use of nitrogen fertilizers like urea; a product of petroleum industry is also readily available in some countries, especially in the Arabic Gulf countries.^12,13^ Urea remains the solid nitrogen fertilizer in common use with the highest nitrogen content and with the lowest transportation costs per unit of nitrogen nutrient. Its quality and ability to fertilize soil for crop cultivation make it one of the most widely used nitrogen fertilizer showing an increase in demand in agriculture. Bioaugmentation/biostimulation of soils with ureolytic bacteria in areas with nitrogen-poor soils has a beneficial side effect of biomineralization.

The aim of the research presented in this paper is to characterize the soil bacterial populations exhibiting ureolytic activity and investigate their diversity and distribution in a region with harsh weather conditions. The innovation resides in the establishment of a screening program of isolated bacteria based on rapid estimation of urease activity and bio-modification of soil. Furthermore, this study addresses the current knowledge gaps related to the microscale response of bio-mediated soils. The objectives of this research include (1) isolation of ureolytic bacteria from the soil in Qatar; (2) identification and differentiation of the isolated strains, using molecular techniques (16S rRNA sequencing) and MALDI-TOF MS (proteins profiling), to study their biodiversity; (3) quantification of the specific production of urease enzyme produced by ureolytic bacteria and (4) analysis of their role in microbially-induced calcite precipitation (MICP) using SEM-EDS technique.

## 2. Methodology

### 2.1. Soil sampling

Soil samples were collected from six locations around Qatar; Hamad Airport area, Duhail, Al-Wakra, Mesaieed, Dukhan and Abu hamur. The samples were stored in ice for transporting to the lab.

### 2.2. Enrichment and isolation of ureolytic bacteria

#### 2.2.1. Enrichment of ureolytic bacteria

Sterile syringe barrels of 30 mL were filled with soil and enrichment medium (g L^−1^): (0.25 glucose, 13.8 sodium acetate, 0.5 Bacto yeast extract and 20 filtered urea) up to 20 mL of volume for each sample. The enrichment solution was modified from the method used by Burbank *et al.* (2012).^5^ A tube was connected to each syringe and was closed by clamp to prevent any flow of solution. After incubation for 3 days, the solution was drained and re-treated for 48 h. Approximately 1 mL of effluent was collected from each syringe into centrifuge tubes. Upon centrifugation at 14 000 rpm for 5 min, pellets were obtained. The pellets were washed with cold sterile phosphate-buffered saline solution (g L^−1^) (8 NaCl, 0.2 KCl, 1.44 Na_2_HPO_4_ and 0.24 KH_2_PO_4_) thrice followed by centrifugation. The resulting pellets were suspended in 5 mL sterile PBS solution.

#### 2.2.2. Isolation of ureolytic bacteria

Serial dilutions of the suspensions obtained through enrichment cultures were carried out and were plated on modified urea agar (g L^−1^) (5 NaCl, 2 KH_2_PO_4_, 1 glucose, 0.012 phenol red indicator, 0.2 peptone, 30 agar and 20 filtered urea) with a final pH of 6.8 as described by Burbank *et al.* (2012)^5^ with modifications. The plates were incubated in dark at 37 °C. The change in color to pinkish red determined an increase of pH. The colonies that successfully changed the color were transferred to sterile tubes with enrichment solution and were kept in rotatory shaker set at 37 °C until turbidity in tubes is observed. The medium was then centrifuged to obtain bacteria pellets which were washed with sterile PBS solution and stored for further microbiological investigations. Purification of each strain was performed with six consecutive sub-culturing followed by preservation at −80 °C in urea medium with 30% glycerol.

### 2.3. Identification of isolates by MALDI-TOF-MS

#### 2.3.1. Sample preparation

The ethanolic/formic acid extraction procedure^26^ was followed to extract proteins. A loop of cells grown overnight were suspended in 300 μL of water. 900 μL of ethanol was added followed by centrifugation at 12 000 rpm for 2 min. The pellet was suspended in 70% formic acid and 100% acetonitrile. The supernatant was transferred to Biotarget plate and was overlaid with 1 μL of HCCA (α-cyanohydroxycinnamic acid) matrix solution.

#### 2.3.2. Analysis of result

The identification was carried out by the Bruker Biotyper software by comparison of spectra within database.^22^ The bacterial profile matching is expressed using log scale from 0.00–3.00 for interpretation of results.

### 2.4. Molecular identification of ureolytic bacteria

Pure cultures of isolates were obtained after overnight growth on Luria Broth (LB) agar medium. Thermal lysis (suspension in 0.5 mL H_2_O, boiling for 10 min, centrifugation for 10 min at 13 000 rpm) was performed on the cells for DNA extraction. The resulting supernatant was used for PCR amplification. Primer RibS73 and RibS74 were used to amplify 16S rRNA fragment.^2^ Purification was done after successful amplification and the amplicons were sequenced by Applied Biosystems 3500 Series Genetic Analyzer System.

BLAST was used to find identical or related sequences in the Gene Bank database provided at NCBI (National Center for Biotechnology Information).

### 2.5. Determination of urease activity of isolates

#### 2.5.1. Sample preparation

20 mL of urea media were inoculated with suspension of bacteria starting with an initial optical density at 600 nm of 0.1. The cultures were incubated at 37 °C in a shaker set at 150 rpm during the period indicated with results. Different urea concentrations were used ranging from 5–50 g L^−1^.

At the time of analysis, samples from each culture were centrifuged for 10 min at 13 000 rpm and the supernatant served for ureolytic activity measurement.

#### 2.5.2. Measurement of urease activity by modified phenol-hypochlorite assay

Phenol-hypochlorite assay^5^ with modifications was used to determine the urease activity by measuring ammonia. 200 μL of culture supernatants were added to 1800 μL of urease buffer (1 mM EDTA, 50 mM HEPES and 20 g L^−1^ filtered urea) and was incubated at 30 °C for 10 min. The control was performed with the sample solutions, but initially they were all placed for at least 30 min in an ice bath. The controls were incubated at −20 °C for 10 min. 200 μL of phenol nitroprusside solution (g L^−1^) (70 phenol and 0.34 nitroprusside, stored at 4 °C in a dark bottle) was added to the mixtures. In the negative control, urease activity is inhibited by cold, and possible positive “urease activity” is then counted as due to ammonia, initially present in the culture medium supernatant, produced by the incubated strain. The blank was performed with all the mixture compounds, except the culture supernatant replaced by urease buffer, to eliminate the potential free ammonia in the urea preparation. Immediately after adding 200 μL of phenol nitroprusside, 200 μL of freshly prepared sodium hypochlorite solution (g L^−1^): (17.5 NaOH and 59.45 Na_2_HPO_4_, 200 mL of bleach, final pH of 10.2; stored at 4 °C in a dark bottle) was added and the mixture was incubated at 30 °C for 20 min. Then, absorbance was measured at 640 nm. The experiments were performed in triplicates. The urease activity is defined as the arbitrary urease activity (AUA). It is the quantity of urease enzyme, responsible for producing 1 μmole min^−1^ of NH_4_^+^ at the experimental conditions (incubation for 10 min at 30 °C). Ammonia standard calibration curve was made by preparing serial dilutions of a 5 × 10^−4^ M stock solution of ammonium chloride.

### 2.6. Scanning electron microscopy (SEM-EDX)

Scanning electron microscopy (SEM) is a technique that uses beam of electrons with high energy to generate different signals on the surface of specimens. The signals reveal the information about the sample under observation such as its morphology, chemical nature, and material orientation. SEM-EDX was used in our research to analyse the morphology and composition of calcium carbonate precipitates.

The precipitates were obtained in 20 mL cultures performed with urea media containing (g L^−1^): 20 urea, 3.7 CaCl_2_, inoculated with separate isolates with an initial OD (600 nm) of 0.1, and incubated at 37 °C in a rotary shaker set at 150 rpm for 30 days. Cultures with both soil (0.4 g) and without soil were inoculated with bacteria. Three controls were performed with the same volume of 20 mL: first, urea medium with calcium chloride, second, 0.4 g soil in urea media with calcium chloride and third, 0.4 g soil in water. The soil used was collected from Abu hamur region in Doha City (Qatar). Precipitates were washed thrice with distilled water and the pellet obtained was oven-dried at 40 °C for 72 h before SEM-EDX analysis.

### 2.7. Statistical analysis

The statistical analysis was done for the results obtained using Microsoft Excel 2016 and ANOVA was also used choosing 95% confidence level.

## 3. Results

### 3.1. Isolation of ureolytic bacteria from soil samples

The basis of this work was isolation of indigenous ureolytic bacteria and evaluation of their growth and production of ureolytic activity in soil surface to determine their potential viable bio-modification processes in Qatari soil with its environmental conditions. Six regions around Doha city were chosen to investigate and evaluate the occurrence and abundance of bacteria which demonstrate ureolytic activities. The samples were collected and enrichment cultures were performed for boosting growth of the corresponding bacteria as mentioned in the Material and Methods section. Ureolytic bacteria were grown in the media (enrichment medium, urea modified agar, urea broth media). Table 1 shows the origin of the sample, the isolates codes and the positive ureolytic ones, using the selective ureolytic medium. Modified urea agar was used to test qualitatively the isolates for their ureolytic activity. The pinkish/red change in colour indicates secretion of urease enzyme which hydrolyses urea, allowing further growth of the isolate on such medium. Out of 30 isolates, 18 isolates were tested positive for ureolytic activity at the experimental conditions.

**Table 1:** Sample origin, isolate codes and positive ureolytic ones for urease activity

Sample origin	Isolate code	Positive for ureolysis
Airport area	QBB1, QBB2, QBB3, QBB4, QBB5, QBB6	QBB1, QBB4, QBB5, QBB6
Duhail	QBB7, QBB8, QBB9, QBB10, QBB11, QBB12, QBB13, QBB14	QBB7, QBB8, QBB11, QBB12, QBB13, QBB14
Al-Wakra	QBB15, QBB16, QBB17, QBB18	QBB17, QBB18,
Mesaieed	QBB19, QBB20, QBB21, QBB22, QBB23, QBB24, QBB25	QBB20, QBB21, QBB22, QBB24, QBB25
Dukhan	QBB26, QBB27, QBB28, QBB29	QBB29
Abu hamur	QBB30	—

### 3.2. Identification of isolates by MALDI-TOF MS

MALDI-TOF MS technique was used to identify the strains exhibiting ureolytic activity. The isolate code, reliability score and the corresponding identity are shown in Table 2. The profile matching was expressed on a log scale from 0 to 3 score. The score was interpreted as per the manufacturer's instruction in which 2.3–3.000 score indicates highly probable species-level identification, 2.00–2.299 shows genus identification and probable species-level identification and 1.70–1.999 score is designated as probable genus level identification. The calibration was done using Bacterial Test Standards (Item Catalogue #255343) provided by the manufacturer (Bruker Daltonics, Bremen, Germany).

**Table 2:** Identification of the isolates by MALDI-TOF MS and identity scores

Isolate code	Score	Identity
QBB1	1.70	*B. subtilis*
QBB4	2.27	*B. cereus*
QBB5	2.20	*B. cereus*
QBB6	2.17	*B. cereus*
QBB7	2.01	*B. cereus*
QBB8	2.00	*B. licheniformis*
QBB11	2.15	*B. cereus*
QBB12	2.21	*B. cereus*
QBB13	2.10	*B. cereus*
QBB14	2.07	*B. cereus*
QBB17	2.10	*B. cereus*
QBB18	2.19	*B. cereus*
QBB20	1.88	*B. licheniformis*
QBB21	2.10	*B. cereus*
QBB22	2.19	*B. cereus*
QBB24	2.18	*B. cereus*
QBB25	2.14	*B. cereus*
QBB29	2.16	*B. cereus*

Most of the isolates were *Bacillus cereus*, one isolate was identified as *Bacillus subtilis* (code: QBB1) with a low identification score, and two were identified as *Bacillus licheniformis* (code: QBB8, QBB20) with high reliability scores. Despite several trials, the identification scores for all the isolates were mostly higher than 2 which can be considered as Probable Species-Level identification.

### 3.3. Differentiation of the isolated strains using protein profiles

**Fig. 1:**
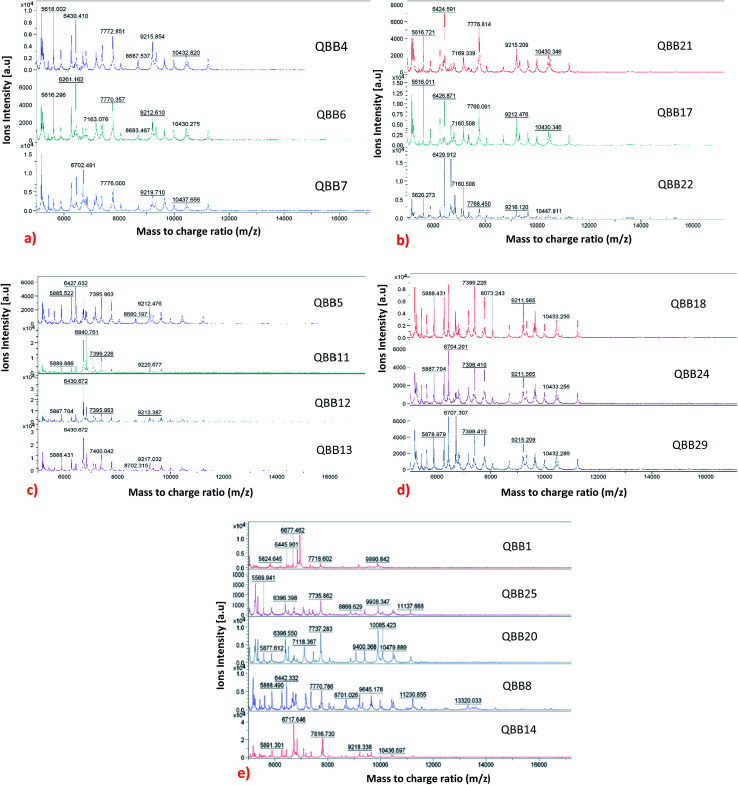
Categorization of isolates into 5 groups based on their similarities and differences in protein profiles: (a) category A; (b) category B; (c) category C; (d) category D and (e) uncategorized.

The MADLI-TOF MS technique was also used for subtyping the strains based on their proteins profiles. Although the technique has been widely used for genus and species level identification, its use for subspecies level identification has not yet been fully explored.^25^ Recently, this technique has been used for subtyping bacteria. The subspecies level identification is quite challenging as the strains (within same species) tend to be very similar in terms of genotype and phenotype.^25^ Lately, subspecies level typing using MALDI-TOF MS has been reported for bacteria like *Yersinia enterocolitica*,^24^ methicillin-resistant *Staphylococcus aureus*,^29^*Staphylococcus enterica*^10^ and *Streptococcus agalactiae*.^18^ In this research, strain differentiation, that relies on the presence or absence of protein peaks^25^ was adopted through proteins profiling. An adequate amount of stable mass signals for the ribosomal proteins typically 2000 to 20 000 Da were obtained. The mass signals are used to produce profile spectra which comprises series of peaks that are conserved at genus, species and even subspecies levels.^6^ The variation among the strains can be observed by the absence or presence of peaks as shown in Fig. 1 for the isolated bacteria. It is evident that some isolates which were identified as same species demonstrated differences at the protein level. The isolates were categorized into five groups (named as category A, B, C, D and un-categorized) based on similarities observed in their protein profiles. The un-categorized group comprises isolates QBB1, QBB8, QBB14, QBB20 and QBB25 that could not qualify for any of the remaining four groups due to the differences in *m*/*z* ratio of their proteins. Fig. 1 shows the comparison of all isolated bacteria belonging to dissimilar categories. Category A comprises isolates QBB4, QBB6 and QBB7 grouped together as they share the same protein peaks at *m*/*z* of 5618.002, 6430.410, 7772.851, 9212.610 and 10 432.820. Similarly, category B isolates includes QBB17, QBB21 and QBB22 sharing same peaks at *m*/*z* of 5616.721, 6426.591, 7169.339, 7776.814, 9215.209 and 10 430.346. Furthermore, isolates under category C include QBB5, QBB11, QBB12 and QBB13. These strains showed similarities in proteins peaks with *m*/*z* of 5885.522, 6430.672, and 9212.476. Lastly, strains QBB18, QBB24 and QBB29 were categorized under category D (Fig. 1) showing peaks at *m*/*z* of 5888.431, 7399.226, 9211.565 and 10 433.256. Variations among the proteins profiles of isolates grouped differently is obvious. For example, presence of peaks at *m*/*z* of 5824.645 and 9890.842 from category A are not found in the other groups. From group B, the peak at *m*/*z* of 6430.410 is not present in group D and un-categorized isolates. Furthermore, peak at *m*/*z* 10 430.346 from group C is not found in groups A, B and D.

In term of occurrence and distribution of the isolates based on their corresponding soil sampling site (Table 1 and Fig. 1), isolates from the Hamad Airport soil sample are distributed in the un-categorized group (QBB1), group A (QBB4 and QBB6) and group C (QBB5). Isolates from Duhail soil belong to un-categorized group (QBB8, QBB14 and QBB), group A (QBB7), and group C (QBB11, QBB12 and QBB13). Al-Wakra soil provided isolates belonging to group B (QBB17) and group D (QBB8). Strains isolated from Mesaieed soil are distributed in un-categorized group (QBB20), group B (QBB21 and QBB22), and group D (QBB24). From Dukhan soil, only one isolate exhibited detectable urease activity, which belongs to group D. Isolates from Abu-Hamur soil did not show ureolytic activity. These results show that some strains could be more distributed around the city than others.

### 3.4. Kinetics of ureolytic activity production and optimization of urea concentration for bacterial growth

**Fig. 2:**
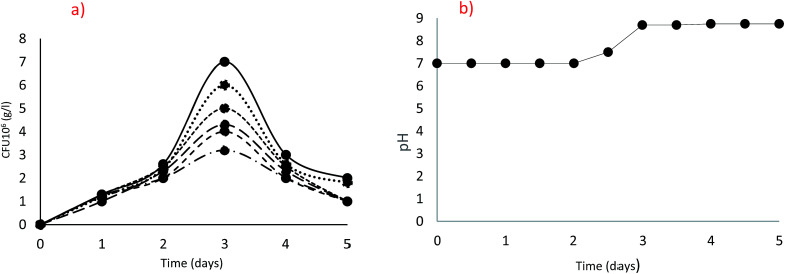
Optimization of urea concentration [5 g L^−1^ (⋯), 10 g L^−1^ (●), 20 g L^−1^ (—), 30 g L^−1^ ( <svg xmlns="http://www.w3.org/2000/svg" version="1.0" width="37.000000pt" height="16.000000pt" viewBox="0 0 37.000000 16.000000" preserveAspectRatio="xMidYMid meet"><metadata> Created by potrace 1.16, written by Peter Selinger 2001-2019 </metadata><g transform="translate(1.000000,15.000000) scale(0.014583,-0.014583)" fill="currentColor" stroke="none"><path d="M80 440 l0 -40 320 0 320 0 0 40 0 40 -320 0 -320 0 0 -40z M880 440 l0 -40 320 0 320 0 0 40 0 40 -320 0 -320 0 0 -40z M1680 440 l0 -40 320 0 320 0 0 40 0 40 -320 0 -320 0 0 -40z"/></g></svg> ), 40 g L^−1^ ( <svg xmlns="http://www.w3.org/2000/svg" version="1.0" width="32.333333pt" height="16.000000pt" viewBox="0 0 32.333333 16.000000" preserveAspectRatio="xMidYMid meet"><metadata> Created by potrace 1.16, written by Peter Selinger 2001-2019 </metadata><g transform="translate(1.000000,15.000000) scale(0.014583,-0.014583)" fill="currentColor" stroke="none"><path d="M960 600 l0 -40 -40 0 -40 0 0 -80 0 -80 40 0 40 0 0 -40 0 -40 80 0 80 0 0 40 0 40 40 0 40 0 0 80 0 80 -40 0 -40 0 0 40 0 40 -80 0 -80 0 0 -40z M80 440 l0 -40 320 0 320 0 0 40 0 40 -320 0 -320 0 0 -40z M1360 440 l0 -40 320 0 320 0 0 40 0 40 -320 0 -320 0 0 -40z"/></g></svg> ), 50 g L^−1^ (----)]; (a) Colony Forming Units (cfu), (b) pH.

In order to compare the isolates for their potential to grow and produce ureolytic activity, the behaviour of each strain as kinetic of growth and activity production should be identified. Since, the optimal conditions, nutrient availabilities and balances are important for efficient microbial activity and may vary from one isolate to another based on its specific metabolism, comparisons between the isolates were performed at the corresponding optimal conditions. All the 18 ureolytic isolates were following a similar pattern of growth, with the highest growth at 20 g L^−1^ urea after 72 h incubation as clearly shown in Fig. 2(a) for the isolate *B. cereus* QBB29. A drastic decrease in the counted cells was noticed beyond that incubation time, independently on the urea concentration. The pH in the cultures started to increase from day 2 and a high pH of 8.75 was observed on day 3. This means the hydrolysis of urea was maximum thereby increasing the pH as can be seen in Fig. 2(b). This may explain the inhibition of growth by excess of substrate (urea) or catabolic repression or increase of pH in the microenvironment of the cell.

### 3.5. Potentialities of production of urease activity by the ureolytic isolates

In order to study the ureolytic bacteria diversity around the studied city, another criterion of differentiation between the isolates belonging to the same group and among the groups differentiated through MALDI-TOF MS analysis, was the determination of their potential to produce ureolytic activity (Table 3). This can be attributed to the level of gene expression and the metabolic regulations in these isolates. Thus, the isolate showing more ureolytic activity than the member of its own group may have expressed the urease gene higher than the others. Similarly, the activity is a result of the total metabolism of the cell, through interactions and regulations occurring in the cell.^15^

**Table 3:** Arbitrary urease activity (AUA) and its specific production of isolates

Categories	Sample locations	Isolate	Identity	cfu (10^6^ cfu mL^−1^)	Arbitrary urease activity (AUA mL^−1^)	Specific production (AUA/10^7^ cfu)	95% confidence interval
A	Airport	QBB4	*B. cereus*	27 ± 1	14.14 ± 0.128	5.2388	0.630 ± 0.114
	Airport	QBB6	*B. cereus*	34 ± 1	8.49 ± 0.086	2.4984	0.378 ± 0.125
B	Mesaieed	QBB21	*B. cereus*	44 ± 3	4.75 ± 0.044	1.0796	0.211 ± 0.037
	Mesaieed	QBB22	*B. cereus*	57 ± 3	7.48 ± 0.084	1.3133	0.333 ± 0.029
C	Airport	QBB5	*B. cereus*	29 ± 9	14.77 ± 0.126	5.094	0.658 ± 0.204
	Duhail	QBB11	*B. cereus*	30 ± 1	7.55 ± 0.085	2.5176	0.336 ± 0.053
	Duhail	QBB12	*B. cereus*	26 ± 5	10.92 ± 0.106	4.1985	0.486 ± 0.078
	Duhail	QBB13	*B. cereus*	35 ± 1	11.52 ± 0.110	3.2918	0.513 ± 0.172
D	Al-Wakra	QBB18	*B. cereus*	35 ± 6	8.13 ± 0.093	2.3245	0.362 ± 0.009
	Mesaieed	QBB24	*B. cereus*	27 ± 5	9.73 ± 0.104	3.6029	0.433 ± 0.132
	Dukhan	QBB29	*B. cereus*	27 ± 6	22.75 ± 0.148	5.0566	1.014 ± 0.667
Un-categorized	Airport	QBB1	*B. subtilis*	20 ± 1	3.05 ± 0.02	1.5231	0.135 ± 0.012
	Duhail	QBB8	*B. licheniformis*	26 ± 2	5.74 ± 0.056	2.2064	0.255 ± 0.044
	Duhail	QBB14	*B. cereus*	37 ± 9	8.05 ± 0.086	2.1746	0.358 ± 0.156
	Mesaieed	QBB20	*B. licheniformis*	45 ± 3	5.44 ± 0.053	1.21005	0.242 ± 0.038

Growth and urease activity determination was performed in the cultures using 20 g L^−1^ urea after 3 days incubation. Statistically significant variations of growth were shown among most of the bacteria as studied with ANOVA analysis with 95% confidence level, showing the diversity of their metabolic requirements and thus metabolites balances which in turn significantly affected the ureolytic activities. Indeed, the highest growth is not necessarily translated into high ureolytic activity in the medium. The cfu of isolate *B. cereus* QBB22 was the highest (57 ± 3 × 10^6^ cfu mL^−1^) but the corresponding AUA was one of the lowest (7.48 ± 0.084). The determination of the specific production of AUA by each isolate would be the criterion of selection of the appropriate one for further applications. Consequently, QBB4, QBB5, QBB12, QBB29 and to some extent QBB13 and QBB24 seemed to be appropriate overproducers of urease activity at the experimental conditions. Some isolates such as QBB7, QBB9, QBB17 and QBB25 were not growing efficiently in liquid and solid modified urea media, therefore, they could not qualify for the determination of the specific production of AUA and be compared to the most efficient isolates. These results showed obvious diversity among the collection of the 18 isolates although 16 are identified as *B. cereus*.

### 3.6. Identification by 16S rRNA ribotyping

In order to combine techniques of identification, QBB4, QBB5, QBB12 and QBB29 exhibiting high urease activity and QBB11 and QBB18 exhibiting relatively low activity were identified. The objective was to confirm and validate the identification performed by MALDI-TOF MS technique.

Table 4 shows the isolate code, its identity, accession number and similarity percentage. There was no discrepancy noted among the results of 16S rRNA and MALDI-TOF MS. Based on these results, it is clear that *Bacillus* sp., especially *B. cereus* are the most abundant bacteria exhibiting urease activity in Qatari soils.

**Table 4:** Identification of 6 isolates by 16S rRNA sequencing

Isolate	Identity	Accession number	Identity percentage	GenBank number for submitted sequences
QBB4	*Bacillus cereus*	KP743133.1	99%	MG745362
QBB5	*Bacillus cereus*	KB743133.1	100%	MG745365
QBB11	*Bacillus cereus*	KY316447.1	96%	MG745366
QBB12	*Bacillus cereus*	CP011151.1	99%	MG745367
QBB18	*Bacillus cereus*	LC260003.1	100%	MG745332
QBB29	*Bacillus cereus*	CP011151.1	96%	MG751339

### 3.7. Role of ureolytic bacteria in MICP

All the isolated strains were tested for their potentiality of calcium carbonate precipitation analysed by SEM/EDS. Similar results were obtained with all the ureolytic activity producers, but with incubation periods fluctuating between 1 month and 2 months for the pH to reach a value of 8.5 (results not shown). Table 5 summarizes the EDS analysis results accompanied by the measurement of pH with 60 days incubated cultures of QBB5, which was shown as one of the best AUA producer exceeding the specific production of 5 AUA/10^7^ cfu (Table 3). In the negative controls performed abiotically (without inoculation of any of the isolates) mineral formation was not detected without soil, while no significant differences were observed in abiotic soil-cultures compared to biotic soil-cultures at the time of inoculation.

**Table 5:** EDS analysis; atomic percentages of carbon, oxygen, calcium and phosphorus in cultures with urea media, calcium chloride and soil (presence/absence) and pH after 60 days incubation of cultures

Medium	QBB5 inoculation	Precipitate type	Composition of precipitates (at%)				pH (after incubation)
			C	O	Ca	P	
Urea medium + CaCl_2_ without soil	No	NA	NA	NA	NA	NA	7.0
Urea medium + CaCl_2_ without soil	Yes	Amorphous CaCO_3_	16.8	65.3	16.8	1.8	8.5
Soil in water	No	No clear amorphous nor crystals of CaCO_3_	7.8	57.1	2.5	0.6	7.0
Urea medium + CaCl_2_ with soil	No	No clear amorphous nor crystals of CaCO_3_	7.8	57.1	2.5	0.6	7.0
Urea medium + CaCl_2_ with soil	Yes	Calcite	24.0	60.5	23.9	1.1	8.8
	Yes	Amorphous CaCO_3_	13.9	60.8	13.4	2.6	8.4

**Fig. 3:**
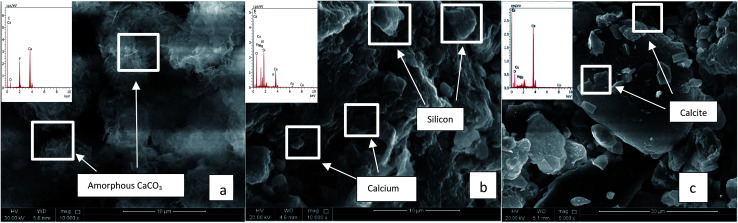
SEM analysis of cultures with urea media. (a) Urea media + CaCl_2_ + QBB5. (b) Soil + urea media + CaCl_2_ without QBB5. (c) Soil + urea media + CaCl_2_ with QBB5.

QBB5 was able to induce formation of amorphous CaCO_3_ precipitates with equal C and Ca proportions (Atomic (at) %). Oxygen (65.31 at%) in the precipitates was much higher than that should be (50.4 at%) in the formed calcium carbonates normally containing 3 times C at% C than Ca (at%). Phosphorus was of 1.8 at% which may be a result of exopolymeric substances precipitated with the minerals. The EPS are also a source of oxygen in the precipitates, explaining the high oxygen content (at%) in the precipitates. Induction of precipitation was concomitant with the increased pH, to almost 8.5, favourable to biomineralization. Fig. 3(a) shows the amorphous calcium carbonate precipitates obtained in urea medium supplemented with calcium chloride and inoculated with QBB5. When soil was added to the urea medium containing calcium chloride, SEM analysis did not show precipitates or crystals of calcium carbonates in abiotic cultures (not inoculated) (Fig. 3(b)). Similar images were obtained with soil in water. However, EDS analysis showed presence of calcium, carbon, oxygen and phosphorus in the used soil in water or in urea medium supplemented with calcium chloride. They are components of soil, as minerals, but not necessary pure calcium carbonate as amorphous or crystal forms. The isolate QBB5 induced calcium carbonate precipitation since clear calcite crystals were observed in the SEM images (Fig. 3(c)). The carbon, oxygen, calcium and phosphorus with atomic percentages of 24.0%, 60.50%, 23.90% and 1.1% were observed in EDS analysis. Although calcium and carbons are in the same proportion (24 at%), it is not accurate to conclude about the importance of the calcium carbonate formation in the soil. The pH of the biotic culture was almost 8.8, highly appropriate for calcium carbonate precipitation. On the other hand, at a different region within the same culture, the carbon, oxygen, calcium and phosphorus with atomic percentages 13.9%, 60.8%, 13.4% and 2.6% were observed in EDS analysis, however, no regular calcite has been observed instead amorphous calcium carbonate is seen precipitated.

## 4. Discussion

Qatar has extreme weather conditions that results in a stressful environment affecting the microbial growth and processes.^2^ However, certain micro-organisms such as *Bacillus*, *Sporolactobacillus* and *Sporosarcina* are well known to be adapted to harsh conditions.^11^ In our study, the identified strains exhibiting urease activity were all identified as belonging to the *Bacillus* genus.

It is important to gather information on the bacterial species that are involved in ureolysis and biomineralization specially when there is no preliminary data available within the region. *B. licheniformis*, *B. subtilis* and *B. cereus* were isolated utilizing modified urea agar among which sub-species of *B. cereus* were abundant. *B. cereus* isolated from soil and marine environment are known to hydrolyse urea.^9,17,27,28^ Similarly, *B. licheniformis* is also involved to precipitate calcium carbonate by ureolysis.^16^ All the species that tested positive for ureolytic activity could grow on urea as sole carbon and nitrogen source consistently. The growth remained stable throughout the experimental analysis. Hence, it can be said that the screened and identified group of species are not characteristic of ureolytic bacteria in Qatar but rather represent species that are able to express the gene responsible for production of urease enzyme under the experimental conditions used. Based on the results (Table 1), it can be concluded that ureolytic bacteria are abundant in Qatari soil and further in-depth analysis could reveal more species.

All isolated strains followed a similar pattern of growth when subjected to different concentrations of urea (Fig. 2). Based on the uniformity in results, 20 g L^−1^ of urea was used as a final concentration in further experiments. In the literature, 20 g L^−1^ of urea is used as well for ureolytic assisted biomineralization experiments.^5,14,20,27^ Regarding urease activity, a new technique of arbitrary urease activity was used that allows researchers to determine the specific production per colony forming unit. As per the ANOVA analysis, the urease activity between all the isolated species was not significantly different. However, based on the specific production (AUA/10^6^ cfu), each species can be categorized in groups. There is a possibility that species with lower specific production needs more time to hydrolyse urea as compared to species with higher specific production due to a change in their conditions.^2^

The hydrolysis of urea and the process of calcium carbonate precipitation are directly linked.^23^ Calcium carbonate precipitation is present in the cultures in its regular (calcite) and irregular (amorphous) precipitate types. The SEM and EDS results provide evidence of the precipitation of calcium carbonate in the cultures by the hydrolysis of urea as well as differences in the morphologies of the soil in cultures with and without the inoculum. Based on the atomic percentages observed, it is evident that carbon and calcium percentages are much lower than the percentages observed for the region with calcite precipitation which means not enough carbon and calcium ions were available for proper crystal formation.

It is important to mention here that urea hydrolysis results in the production of ammonia (NH_3_) and carbonic acid (H_2_CO_3_). The carbonic acid further dissociates and produce carbonate ions (CO_3_^−2^). Furthermore, carbonate ions would react with calcium ions (from CaCl_2_) and precipitate calcium carbonate (CaCO_3_) at higher pH.^8^ From this, we can conclude that the precipitation of calcium carbonate in the cultures is the outcome of urea hydrolysis by ureolytic bacteria as no foreign source of carbonate in the cultures was introduced.

The result of this research showed that the abundance of ureolytic *Bacillus* sp. is high with respect to any other genus. There could be a possibility that all the conditions were favourable for this genus to survive and grow as compared to others during the isolation.

## 5. Conclusion

The results obtained from this research confirms the presence of ureolytic bacteria in Qatari soil indicating their adaptation to the harsh environment. Using MALDI-TOF MS technique, differentiation of bacteria within the same species such as *B. cereus* was possible through protein profiles. This showed that the urease activity can be different within the same species, perhaps, due to the differences in the levels of gene expression for urease enzyme. This research also proposed a new method for determining urease activity *i.e.* arbitrary urease activity (AUA). Hence, the isolates categorized by high AUA values demonstrates higher ureolytic activity. Since, the biodegradation of urea by these bacteria will lead to increase in pH, thereby enhancing minerals precipitation such as calcium carbonate. Therefore, SEM-EDX analysis was performed in order to analyse the morphology and composition of deposits. It was noticed that the presence or absence of ureolytic bacteria significantly affects the composition and morphology of calcium carbonate crystals. Accordingly, it can be concluded that the indigenous ureolytic bacteria present in Qatari soil has the potential to carry out soil stabilization in the presence of urea by enhancing biomineralization. Therefore, introduction of urea into the soil can also be one of the management measure to control the soil erosion caused by winds and to enhance soil stabilization in Qatar.

## 6. Author contributions

Nabil Zouari, conceived and designed the experiments, contributed in analysis of the data and wrote the paper. Shazia Bibi and Meriam Oualha, performed the experiments and contributed in writing of the manuscript. Mohammad Yousaf Ashfaq, performed the MALDI-TOF MS experiments and contributed in writing of the manuscript. Muhannad T. Suleiman, contributed in conceiving the experiments, analysis of the data and review of the manuscript.

## Conflicts of interest

The authors declare no conflict of interest. All authors decided to publish this work. The funding sponsors had no role in the design of the study; in the collection, analyses, or interpretation of data; in the writing of the manuscript, and in the decision to publish the results.

## Supplementary Material
